# Hemophagocytic Lymphohistiocytosis in Pregnancy: A Case Series and Review of the Current Literature

**DOI:** 10.1155/2019/9695367

**Published:** 2019-02-12

**Authors:** Jessica Parrott, Alexander Shilling, Heather J. Male, Marium Holland, Cecily A. Clark-Ganheart

**Affiliations:** ^1^Division of Maternal Fetal Medicine, Department of Obstetrics and Gynecology, University of Kansas Medical Center, 3901 Rainbow Boulevard, Kansas City, KS 66160, USA; ^2^Division of Hematologic Malignancies and Cellular Therapeutics, Department of Internal Medicine, University of Kansas Medical Center, 3901 Rainbow Boulevard, Kansas City, KS 66160, USA

## Abstract

**Background:**

Hemophagocytic lymphohistiocytosis (HLH) is a rare disease that can be fatal in pregnancy. We report two cases of severe HLH that highlight etoposide use in pregnancy.

**Case 1:**

28-year-old G2P1 with lupus presented at 18 weeks with acute hypoxic respiratory failure, hepatic dysfunction, leukopenia, thrombocytopenia, and elevated ferritin. Bone marrow biopsy confirmed HLH. Etoposide and corticosteroid treatment was initiated per HLH protocol; however clinical status declined rapidly. Fetal demise occurred at 21 weeks and she subsequently suffered a massive cerebral vascular accident. She was transitioned to comfort measures and the patient deceased.

**Case 2:**

37-year-old G4P3 presented at 25 weeks with fever, acute liver failure, thrombocytopenia, and elevated ferritin. HLH treatment was initiated, including etoposide, and diagnosis confirmed with liver biopsy. Fetal growth restriction was diagnosed at 27 weeks. Delivery occurred at 37 weeks. The neonate was found to be CMV positive despite negative maternal serology.

**Conclusion:**

The addition of etoposide to corticosteroid use is a key component in HLH treatment of nonpregnant individuals. While this is usually avoided in pregnancy, the benefit to the mother may outweigh the potential harm to the fetus in severe cases and it should be strongly considered.

## 1. Introduction

Hemophagocytic lymphohistiocytosis (HLH) is a rare life threatening disease characterized by the over activation of normal T cells and macrophages and the uninhibited release of cytokines leading to a cytokine storm and a self-perpetuating loop of dysfunctional immune system regulation [1, 2]. This process of immune system activation primarily arises from a Th1 cytotoxic response via the release of IFN-*γ*, IL-2, and IL-12 [1]. The over activated macrophages participate in uncontrolled hemophagocytosis of leukocytes, platelets, erythrocytes, and their precursors leading to severe cytopenias. As a result, increased cytokine production of IL-1, IL-6, and TNF-*α* is responsible for the systemic manifestations of the disease including, but not limited to fever, hypertriglyceridemia, hepatic dysfunction, and hypofibrinogenemia [1]. Without prompt recognition and treatment, patients progress to end organ failure [1]. HLH can be a diagnostic challenge. It is exceedingly rare in the pregnant population and the delay in diagnosis and treatment can be devastating for the mother and fetus. Here, we summarize two unique cases that emphasize the importance of keeping a broad differential diagnosis and reiterate the importance of rapid diagnosis and treatment of HLH in the pregnant patient.

## 2. Case 1

A 28-year-old para 1001 woman with a past medical history of systemic lupus erythematosus was found to be 5-week pregnant at the onset of a lupus flare. She reported headaches, fevers, fatigue, and arthralgias. She had a known positive antinuclear antibody (ANA) level of 1:640 as well as positive rheumatoid factor, anti-double stranded DNA antibodies, anti-SSA antibodies, anti-smith antibodies, lupus anticoagulant, and anti-RNP antibodies. The patient was managed in conjunction with rheumatology. The patient was started on hydroxychloroquine 200 mg twice daily and aspirin 81 mg daily. She was scheduled to begin limited ultrasounds every two weeks beginning at 16 weeks due to her positive anti-SSA antibody status. By 8 weeks, she exhibited mouth and lip sores, lymphadenopathy, pleuritic chest pain, and a maculopapular rash. She was found to have a low C3 (30.0) and elevated liver enzymes (AST 141 U/L and ALT 58 U/L) so prednisone 10 mg twice daily was initiated. Despite the prednisone and hydroxychloroquine, her symptoms persisted and due to anorexia and nausea/vomiting of pregnancy, she experienced a 20-pound weight loss over the next 4 weeks. After documenting a normal thiopurine methyltransferase enzyme activity, the patient was started on azathioprine 100 mg daily. Within one week of starting azathioprine the patient's pain considerably decreased and her lymphadenopathy almost resolved.

At 18 5/7 weeks, the patient presented to clinic with new onset shortness of breath and was subsequently admitted to the intensive care unit with acute hypoxic respiratory failure. During the week prior, the patient complained of daily fevers. The patient's respiratory status rapidly declined, requiring intubation and mechanical ventilation. Laboratory studies upon admission were notable for a normal white blood cell (WBC) count of 4.6 K/UL, mild anemia with a hemoglobin 10.3 gm/dL, normal platelet count of 198 K/UL, AST 123 U/L, ALT 57 U/L, and lactate dehydrogenase (LDH) of 110 U/L. A chest X-ray showed five lobe infiltrates and computed tomography (CT) angiography of the chest was negative for pulmonary embolism. An abdominal ultrasound showed mild splenomegaly (12.7 cm in length). She was started on broad spectrum antibiotics; however extensive infectious evaluation including blood, urine, and bronchial cultures were all negative for an infectious process. Within 24 hours, the patient developed leukopenia and thrombocytopenia with WBC 3.1 K/UL and platelets of 60 K/UL. During the course of her initial work-up she was also noted to have a significantly elevated ferritin of 3534 ng/mL. With the negative infectious work-up and lack of response to antibiotics, her acute respiratory distress syndrome (ARDS) was felt to be secondary to an autoimmune etiology and she was started on high dose methylprednisolone.

Given her negative work-up thus far and worsening pancytopenia, hematology was consulted at 19 1/7 weeks. Soluble IL-2 receptor (sCD25) levels were sent for evaluation and later returned as 11,370. A bone marrow biopsy was performed showing hemophagocytosis of all cell lineages and the diagnosis of HLH syndrome was confirmed. She was started on etoposide and dexamethasone per the HLH-94 treatment protocol and she received a 5-day course of intravenous immunoglobulin. Over the next week, the patient continued to deteriorate with progressive pancytopenia (nadirs of WBC 1.8 K/UL, hemoglobin 6.1 gm/dL, and platelets 18 K/UL), persistent fevers, and increasing ferritin (>7500 ng/mL max). Persistent fetal tachycardia was observed daily into the 200s. At 20 4/7 wga, the patient coded twice requiring chest compressions without medications (each episode less than 1 minute in duration). Over the next week there was cyclical improvement and deterioration in the patient's respiratory status. A growth ultrasound was done and intrauterine growth restriction (estimated fetal weight 210 grams) was noted.

At 21 4/7 wga, the patient developed vaginal bleeding and subsequently delivered a demised male fetus. The following day the patient developed tachycardia into the 170s and a temperature of up to 103.0°F. Rapid neurologic decline prompted a head CT which revealed a left middle cerebral artery infarct. Aggressive measures including cyclosporine were attempted; however the patient had further neurologic deterioration and was transitioned to comfort measures. Autopsy was declined by the family.

## 3. Case 2

A 37 yo para 3003 presented to an outside hospital at 24 weeks with fevers, a diffuse pruritic rash, oral lesions, and a frontal headache. She was jaundiced in appearance and was noted to have liver enzymes in the 1000s and a total bilirubin of 11.1 mg/dL. She had a negative work-up for hepatitis, influenza, HIV, measles, mycoplasma, rubella, CMV, EBV, and syphilis. An abdominal ultrasound was unremarkable other than a contracted gallbladder. She was transferred to our institution at 25w2d. By this time, her liver enzymes had improved to AST 983 U/L and ALT 413 U/L. Her total bilirubin, however, remained elevated at 12.2 mg/dL. Additional significant laboratory values included an elevated lactate dehydrogenase 1049 U/L and low platelets 91 K/UL; lactic acid and white blood cell count were within normal limits. Additional investigations into possible infectious or autoimmune etiologies were initiated. Hepatology, infectious disease, rheumatology, and hematology services were consulted. Overnight, the patient continued to have rising liver enzymes (AST 1118 U/L, ALT 437 U/L) and became hypothermic with a normal lactic acid (1.7 mmol/L). The decision was made to transfer her to the medical ICU.

Extensive laboratory work-up was undertaken for the acute liver injury and fever of unknown origin. Hepatology felt the most likely diagnosis was autoimmune hepatitis as her lab abnormalities could be explained by acute liver injury (hemoglobin 12.0 g/dL, platelets 82 K/UL, creatinine 0.55 mg/dL, total bilirubin 12.2 mg/dL, AST 1118 U/L, ALT 437 U/L, direct bilirubin 7.7 mg/dL, fibrinogen 232 mg/dL, ferritin 8110 ng/ml, haptoglobin <30 mg/dL, triglycerides 821 mg/dL, albumin 1.9 g/dL, ammonia 36 mcmol/L, and normal peripheral smear). The patient's blood cultures and infectious work-up were negative. Her autoimmune work-up was positive for an ANA of 1:320 but was otherwise negative. With negative autoimmune labs and the elevated ferritin level, the leading diagnosis was HLH. Soluble interleukin-2 receptor levels were ordered (later resulted as 15,110) and the patient was started on dexamethasone 10 mg/m^2^ daily per the HLH 94 protocol.

On hospital day 4, the patient's clinical picture continued to worsen. Her platelets were 74 K/UL, INR 1.4, fibrinogen 152 mg/dL, total bilirubin 12.4 mg/dL, AST 1207 U/L, and ALT 807 U/L. The patient was started on 75% reduced dose etoposide in addition to the dexamethasone per the HLH 94 protocol and she underwent liver and bone marrow biopsy for definitive diagnosis. The bone marrow biopsy showed normocellular marrow (60%) and was negative for hemophagocytosis. The liver biopsy showed hemophagocytic syndrome with acute liver cell injury. Within 48-72 hours of initiating the etoposide, the patient began to show improvement and she was subsequently transferred out of the ICU.

The patient was treated with two additional doses of etoposide while inpatient. Her course was complicated by the development of steroid-induced gestational diabetes and she was started on subcutaneous insulin for glucose control. Her pregnancy was monitored closely with weekly ultrasounds to monitor fluid and Doppler studies in addition to growth ultrasounds every 4 weeks. At 27 4/7 weeks, the fetus was noted to have intrauterine growth restriction with normal Doppler studies. At that time, her labs had continued to improve and she was discharged from the hospital to complete her therapy as an outpatient.

The remainder of her pregnancy was unremarkable. Her liver function returned to normal over the course of her pregnancy. She was tapered off of the dexamethasone and her gestational diabetes was well controlled. We continued to follow her pregnancy closely with ultrasound and her Doppler studies remained normal, despite the fetal growth restriction. She was subsequently induced at 37 0/7 weeks and had a normal vaginal delivery of a significantly growth restricted male infant weighing 1305 gm. All parameters measured less than the 3rd percentile (head circumference of 28cm) and the infant was given APGARS of 8/9. The infant's neonatal ICU stay was remarkable for a positive quantitative urine CMV PCR (treated with valacyclovir), despite the patient having a negative CMV work-up at the time of her initial presentation. Thus, CMV may have been the inciting cause of this case of HLH. The infant was also noted to have hypothyroidism and was started on synthroid. No other abnormalities were found and the infant was discharged home.

## 4. Comment

Here, we have presented two cases of HLH during pregnancy treated with etoposide. The only current published case that utilized etoposide, presented by Klein* et al.*, was associated with an EBV infection, diffuse gastrointestinal ulcers causing upper and lower gastrointestinal bleeding, disseminated intravascular coagulation (DIC), and hemorrhagic shock ultimately leading to the death of their patient [25]. Even though etoposide is one of the initial treatment methods for HLH outside of pregnancy, it is considered a Pregnancy Category D drug by the United States Food and Drug Administration and its use to treat HLH during pregnancy is very rare. The addition of etoposide to corticosteroid use has been shown to induce prolonged remission of HLH and has become a key component in current, nonpregnancy related, and treatment protocols [26]. The severity of the patient's clinical condition at initiation of treatment in both our cases and the case of Klein* et al.* indicated that the use of etoposide would be of more benefit to the patient than harm to the fetus.

Hemophagocytic lymphohistiocytosis (HLH) occurring in an adult population is overwhelmingly secondary to an underlying cause. Secondary HLH can be associated with infections (Epstein-Barr virus, Cytomegalovirus, and herpes simplex virus), malignancies (lymphomas), and autoimmune diseases (systemic lupus erythematosus; rheumatoid arthritis) [1, 2]. The most common presenting clinical symptoms are fever and hepatosplenomegaly. With a rapidly progressive disease course and rarity of the disease, delay in diagnosis lends to a high mortality rate. Without treatment, the mean survival time is approximately two months [27, 28].

Currently guidelines recommend using criteria established by the Histiocyte Society in 2004 for diagnosis [29]. It is important to note that a positive bone marrow biopsy may not be seen early in the disease course (as in case 2) and repeat bone marrow biopsies should be considered. However, a positive bone marrow biopsy is not required for diagnosis. Due to the direct correlation with increased T-cell and NK-cell activity, Mayama* et al.* suggest that measuring soluble IL-2 receptor levels is the most specific diagnostic criteria for HLH [13]. However, delaying initiation of therapy while waiting for test results to confirm a diagnosis may be detrimental. Therefore, treatment should be started if there is high clinical suspicion.

Recently published results from the HLH-2004 protocol establish that there was no benefit to up front addition of cyclosporine and that induction therapy should remain to be dexamethasone and etoposide [29]. Dosing adjustments and frequency for etoposide based on organ function in secondary HLH is common, and etoposide is often removed from the regimen as patients stabilize. Patients with secondary HLH may have complete eradication of their disease from the use of all, or portions of, the HLH 94 protocol and/or treatment of the underlying infection, malignancy, or autoimmune condition that is driving the dysfunctional immune system. Unfortunately, some of these medications are associated with fetal toxicity, making the treatment of HLH in the pregnant patient exceptionally difficult.

An updated list of cases of HLH diagnosed during pregnancy is presented in Table 1. The cause of HLH in 5 of the 24 presented cases was never determined. Teng* et al. *and Shukla* et al.* present the hypothesis that these cases with unknown etiology may be pregnancy derived from fetomaternal trafficking, similar to the pathogenesis of preeclampsia in which the immature placenta releases genetically foreign material into maternal circulation. Maternal T-lymphocytes, unable to recognize these unfamiliar human lymphocyte antigens, may trigger a systemic inflammatory response and cytokine storm similar to what is seen in HLH [18, 19, 30, 31]. The actual mechanism of pregnancy inducing HLH is still unknown, but some suggest a definite connection between the two secondary to several reports of symptom resolution and disease remission after pregnancy termination (via therapeutic abortion, spontaneous abortion, or cesarean delivery). Systemic lupus erythematosus (5/24), Epstein-Barr virus (3/24), and Parvovirus B19 (3/24) were among the majority of associated diseases. Other triggers seen in individual cases included HIV-1 and malaria, Still's disease, B-cell non-Hodgkin lymphoma, hyperemesis, primary Sjogren's syndrome, pyogenic liver abscess, autoimmune hemolytic anemia (AIHA), cytomegalovirus (CMV), herpes simplex virus 2 (HSV2), and varicella zoster virus (VZV).

Among these cases, the most common and safest method of treatment during pregnancy was high dose corticosteroids and/or directed treatment of the underlying cause of the HLH, if known. Of the 24 cases, corticosteroids were used in 18 of them, usually as initial treatment. The relatively low risk of birth defects in women taking corticosteroids during pregnancy, especially after the first trimester, suggests that the benefit of using these drugs greatly outweighs the risks. Excluding the use of antibiotics, in eight of the cases, corticosteroids were the only treatment methods used. In six of these eight cases, it was believed that remission of the disease was due solely to steroid administration. In the other two cases, it is unclear whether remission was due to steroids or termination of the pregnancy (via spontaneous abortion or cesarean delivery). While antibiotics were initiated in half of the cases (14/24), disease remission was never attributed to the use of antibiotics alone. Other treatment options employed in these cases included IVIg (intravenous immunoglobulin-11/24), cyclosporine A (5/24), antithrombin III (AT III-4/24), antiviral medications (4/24), etoposide (2/24), rituximab (1/24), R-CHOP (rituximab, cyclophosphamide, doxorubicin, vincristine, prednisone chemotherapy regimen-1/24), HAART (highly active antiretroviral therapy-1/24), and splenectomy (1/24) [3–24, 31].

In summary, when constructing the differential diagnosis of a pregnant patient with fever, cytopenias, hepatic dysfunction, and elevated ferritin, it is important to consider hemophagocytic lymphohistiocytosis as a potential etiology. Also, in illnesses where the etiology appears nebulous, consideration to the addition of ferritin to laboratory panels may assist in honing in on the correct diagnosis. Finally, in severe/catastrophic cases, the benefit of etoposide use in pregnancy likely outweighs the potential harm to the fetus and should be strongly considered.

## Figures and Tables

**(a):** 

Case	Age (yrs)	Cause/Associated Diagnoses	Gestation at Presentation (wks)	Hepatomegaly/Splenomegaly/Lymphadenopathy	WBC (K/UL)	Hgb (g/*μ*L)	Plt (K/UL)	ALT/AST (U/L)
Arewa and Ajadi 2011	31	HIV-1 and malaria	21	-/-/-	4.2	5	125	6/4
Chien 2009	28	Unknown	23	NA	8.9	5.9	11	NA/92
Chmait 2000	24	EBV with necrotizing lymphadenitis	29	+/+/+	2.6	9	23	408/371
Dunn 2012	41	Still's disease	19	NA	Neutropenia	9.8	343	2695/1258
Gill 1994	30	Unknown	17	+/+/NA	3.1	8.7	19	-/241
Gonzalez 2008	27	Parvovirus B19	NA	NA	NA	NA	NA	NA
Hanaoka 2007	33	B-cell non-Hodgkin lymphoma	21	+/+/NA	5.8	9.5	104	110/170
Hannebicque-Montaigne 2012	29	SLE	21	NA	NA	NA	NA	NA
Kim 2013	29	SLE	12	+/+/NA	3.8	6.9	94	NL/56
Klein 2014	39	EBV	30	NA	Pancytopenia	Pancytopenia	Pancytopenia	Elevated
Komaru 2013	36	Primary Sjogren's syndrome	38d postpartum	NA	NA	NA	NA	NA
Mayama 2014	28	Parvovirus B19	20	-/-/-	0.6	4.2	83	NL
Mihara 1999	32	EBV	16	NA	NA	NA	NA	NA
Nakabayashi 1999	30	Unknown	21	NA/-/-	2.9	11.1	7.7	-/87
Perard 2007	28	SLE	22	NA/-/-	3.5	9.2	80	-/45
Samra 2015	36	Unknown	16	+/+/-	1.3	9.9	125	NA
Shukla 2013	23	Unknown	10	+/+/-	1.9	6.3	18	NL
Teng 2009	28	AIHA	23	+/+/NA	8.9	4.9	109000	18/92
Tsuda 1995	30	Parvovirus B19	9d postpartum	NA	NA	NA	NA	NA
Tumian 2015	35	CMV	38	-/-/-	15.0	7.1	84	389/341
Yamaguchi 2005	NA	VZV	NA	NA	NA	NA	NA	NA
Yamanaka 1995	23	VZV	NA	NA	NA	NA	NA	NA
Yoshida 2009	33	SLE	After delivery	NA	NA	NA	NA	NA
Case 1	28	SLE	19	-/+/+	3.1	10.3	60	58/141
Case 2	37	Unknown	24	-/-/-	NL	12.0	82	437/1118

*∗*Neonate demised 3-4 days after birth due to respiratory distress.

NA: data not available; NL: normal; HIV-1: human immunodeficiency virus 1; EBV: Epstein-Barr virus; SLE: systemic lupus erythematosus; AIHA: autoimmune hemolytic anemia; CMV: cytomegalovirus; VZV: varicella zoster virus; WBC: white blood cell count; Hgb: hemoglobin; Plt: platelet count; ALT: alanine transaminase; ALT: aspartate transaminase; Bx: biopsy; BM: bone marrow; Abx: antibiotics; IVIg: intravenous immunoglobulin; Tx: treatment; HAART: highly active antiretroviral therapy; AT III: antithrombin III; R-CHOP: rituximab, cyclophosphamide, doxorubicin, vincristine, prednisone chemotherapy regimen; PVSCT: peripheral blood stem cell transplant; CSA: cyclosporine A; G-CSF: granulocyte colony stimulating factor; PTL: preterm labor; HELLP: hemolysis elevated liver enzymes and low platelet count syndrome; Oligo: oligohydramnios; UA: umbilical artery; IUGR: intrauterine growth restriction; TAB: therapeutic abortion; IUFD: intrauterine fetal demise; PROM: premature rupture of membranes; ICH: intracerebral hemorrhage; SAB: spontaneous abortion; C/S: cesarean section; VD: vaginal delivery.

References: Arewa and Ajadi 2011 [3]; Chein 2009 [4]; Chmait 2000 [5]; Dunn 2012 [6]; Gill 1994 [7]; Gonzalez 2008 [8]; Hanaoka 2007 [9]; Hannebicque-Montaigne 2012 [10]; Kim 2013 [11]; Klein 2014 [3]; Komaru 2013 [12]; Mayama 2014 [13]; Mihara 1999 [14]; Nakabayashi 1999 [15]; Perard 2007 [16]; Samra 2015 [17]; Shukla 2013 [18]; Teng 2009 [19]; Tsuda 1995 [20]; Tumian 2015 [21]; Yamaguchi 2005 [22]; Yamanaka 1995 [23]; Yoshida 2009 [24].

**(b):** 

Case	Ferritin (ng/mL)	Triglyceride (mg/dL)	Bone Marrow Bx	Abx	Steroids	IVIg	Etoposide	Other Tx	Complications	Gestation (wks), Delivery Method	Maternal/Fetal Survival
Arewa and Ajadi 2011	NA	NA	+					Amodiaquine, HAART		Term, C/S	NA/Yes
Chien 2009	4517.5	386	+	+	+			Percutaneous nephrostomy tubes	PTL	30, C/S	Yes/No*∗*
Chmait 2000	NA	NA	+	+		+		Acyclovir, AT III	HELLP, oligo, intermittent absent UA diastolic flow	30, C/S	No/Yes
Dunn 2012	3745	358	+	+	+				IUGR	30, C/S	Yes/Yes
Gill 1994	NA	NA	+	+		+				Term, NA	Yes/Yes
Gonzalez 2008	NA	NA	NA	+	+			AT III	PROM	36, NA	Yes/Yes
Hanaoka 2007	587.6	258	+	+	+			R-CHOP, PBSCT	Oligo	29, C/S	Yes/Yes
Hannebicque-Montaigne 2012	NA	NA	NA							NA	NA
Kim 2013	2890	NA	- BM, + spleen	+	+	+		CSA, splenectomy	TAB	14, NA	Yes/No
Klein 2014	Elevated	NA	- BM, + jejunal		+		+	CSA, rituximab		31, C/S	No/Yes
Komaru 2013	NA	NA	NA		+					NA	Yes/Yes
Mayama 2014	1269.2	NA	+		+					37, VD	Yes/Yes
Mihara 1999	NA	NA	NA		+	+		Acyclovir, gabexate mesilate		35, NA	Yes/Yes
Nakabayashi 1999	7240	NA	+	+		+		AT III	Preeclampsia, IUGR, Oligo	29, NA	Yes/Yes
Perard 2007	15000	970	+	+	+	+			PROM, Eclampsia, PTL, ICH	30, NA	Yes/Yes
Samra 2015	4000	110	-	+	+					NA, VD	Yes/Yes
Shukla 2013	>2200	588	+		+				SAB	NA	Yes/No
Teng 2009	8926	386	+		+					29, C/S	Yes/No*∗*
Tsuda 1995	NA	NA	NA							NA	NA
Tumian 2015	NA	NA	+	+	+	+		Plasma exchange, CSA, ganciclovir		38, C/S	NA
Yamaguchi 2005	865.8	258	+	+	+	+		Acyclovir, CSA		37, C/S	Yes/Yes
Yamanaka 1995	NA	NA	NA	+	+	+		AT III, mesilate G-CSF	Preeclampsia	36, NA	Yes/Yes
Yoshida 2009	NA	NA	NA		+					NA	Yes/Yes
Case 1	4573	269	+	+	+	+	+	CSA, azathioprine, hydroxychloroquine	Abruption, IUFD, stroke	21, VD	No/No
Case 2	8110	821	- BM, + liver		+		+			37, VD	Yes/Yes

*∗*Neonate demised 3-4 days after birth due to respiratory distress.

NA: data not available; NL: normal; HIV-1: human immunodeficiency virus 1; EBV: Epstein-Barr virus; SLE: systemic lupus erythematosus; AIHA: autoimmune hemolytic anemia; CMV: cytomegalovirus; VZV: varicella zoster virus; WBC: white blood cell count; Hgb: hemoglobin; Plt: platelet count; ALT: alanine transaminase; ALT: aspartate transaminase; Bx: biopsy; BM: bone marrow; Abx: antibiotics; IVIg: intravenous immunoglobulin; Tx: treatment; HAART: highly active antiretroviral therapy; AT III: antithrombin III; R-CHOP: rituximab, cyclophosphamide, doxorubicin, vincristine, prednisone chemotherapy regimen; PVSCT: peripheral blood stem cell transplant; CSA: cyclosporine A; G-CSF: granulocyte colony stimulating factor; PTL: preterm labor; HELLP: hemolysis elevated liver enzymes and low platelet count syndrome; Oligo: oligohydramnios; UA: umbilical artery; IUGR: intrauterine growth restriction; TAB: therapeutic abortion; IUFD: intrauterine fetal demise; PROM: premature rupture of membranes; ICH: intracerebral hemorrhage; SAB: spontaneous abortion; C/S: cesarean section; VD: vaginal delivery.

References: Arewa and Ajadi 2011 [3]; Chein 2009 [4]; Chmait 2000 [5]; Dunn 2012 [6]; Gill 1994 [7]; Gonzalez 2008 [8]; Hanaoka 2007 [9]; Hannebicque-Montaigne 2012 [10]; Kim 2013 [11]; Klein 2014 [3]; Komaru 2013 [12]; Mayama 2014 [13]; Mihara 1999 [14]; Nakabayashi 1999 [15]; Perard 2007 [16]; Samra 2015 [17]; Shukla 2013 [18]; Teng 2009 [19]; Tsuda 1995 [20]; Tumian 2015 [21]; Yamaguchi 2005 [22]; Yamanaka 1995 [23]; Yoshida 2009 [24].
